# Assessing reservoir host status in leishmaniasis with special reference to the infectiousness of *Leishmania (Viannia) braziliensis* infections in wild rodents*

**DOI:** 10.1590/0037-8682-0503-2023

**Published:** 2023-12-15

**Authors:** Jeffrey J. Shaw, José F. Marinho-Júnior, Orin Courtenay, Sinval P. Brandão-Filho

**Affiliations:** 1 Universidade de São Paulo, Instituto de Ciências Biomédicas, Departamento de Parasitologia, São Paulo, SP, Brasil. Universidade de São Paulo Instituto de Ciências Biomédicas Departamento de Parasitologia São Paulo SP Brasil; 2 Fundação Oswaldo Cruz, Instituto Aggeu Magalhães, Recife, PE, Brasil. Fundação Oswaldo Cruz Instituto Aggeu Magalhães Recife PE Brasil; 3 University of Warwick, School of Life Sciences and the Zeeman Institute, Coventry, United Kingdom. University of Warwick School of Life Sciences and the Zeeman Institute Coventry United Kingdom

**Keywords:** Cutaneous leishmaniasis, Xenodiagnosis, Reservoir host, Molecular xenodiagnostic surrogate, Asymptomatic infections

## Abstract

Molecular methods have been responsible for a notable increase in the detection of Leishmaniinae infections in wild animals. Determining their infectiousness is of paramount importance in evaluating their epidemiological significance. One of the most efficient ways of determining infectiousness for vector borne diseases is xenodiagnosis with the appropriate vector. However, this is logistically very difficult to accomplish in the field, and an ideal solution is to find a molecular surrogate for xenodiagnosis. In this review we discuss different approaches to the problem by focusing on the infectiousness of *Leishmania* (*Viannia*) *braziliensis* in rodents under laboratory and field conditions. Comparisons with similar studies for other *Leishmania* species emphasizes that there are pivotal differences in the infectiousness and the importance of asymptomatic infections in different hosts. Potentially the most promising surrogate is the real time quantitative PCR (qPCR). However, its success depends on choosing a tissue that relates to the vector’s feeding location and the parasite’s tissue tropism. This requires detailed knowledge of the infection of each species in its wild hosts. We conclude that for *L*. (*V*.) *braziliensis* infections in wild rodents the tissue of choice for a molecular xenodiagnostic test, based on the qPCR is blood, providing that a significant number of samples must be examined.

## INTRODUCTION

Leishmaniasis is a vector-borne disease caused by protozoan parasites of the subfamily Leishmaniinae^1^. The responsible parasites are transmitted between animals and to man by the bites of infected female phlebotomine and culicoidini midges that are both pool feeders. The relative importance of a vertebrate species as a source of infection in leishmaniasis depends on a combination of factors from its behaviour to its interactions with the parasite, vector, and human population. Criteria have periodically been suggested and reviewed^2^^,^^3^^,^^4^^,^^5^^,^^6^ in attempting to evaluate more accurately an animal’s relative importance in the disease’s transmission cycle. Delineating the importance of infections found in wild animals is complex but is crucial for effective disease control.

## RESERVOIR HOST

Ashford^7^^,^^8^ suggested the terms “primary reservoir” (*R*_*0*_ *>* 1) used for a host that perpetuates the enzootic cycle and “secondary reservoir” (*R*_*0*_ *<* 1) that does not maintain the enzootic but contributes to the cycles transmission potential in a multi-host scenario. Chaves et al.^6^, considered that enzootics are multi-host situations that approximate metacommunities and suggested the use of the terms “Sources and Sinks” used in ecology^9^. Under these concepts primary and secondary reservoirs are sources irrespective of the part they play in the maintenance of the enzootic cycle. Sinks are dead end infections that do not lead to new ones. The multi-host scenario is supported by recent observations that detected *L.* (*V.*) *braziliensis* in 6/8 different rodent species^10^ captured an endemic American Cutaneous Leishmaniasis (ACL) focus. However, the multi-host concept may not apply to all species. For instance, *L.* (*V.*) *naiffi* occurs only in armadillos. This leads us to suggest that another accepted parasitological concept is useful in understanding the importance of infections in wild animals in which the parasites can be considered as either generalists, such as *L.* (*V.*) *braziliensis* or specialists, such as *L.* (*V.*) *naiffi.* Judged by the incidences of the different Leishmaniinae in man generalists are greater public health threats than specialists^11^^,^^12^^,^^13^. 

Below are key factors, some of which are covered in excellent studies that contribute to assessing the importance of an animal as a source of infection in leishmaniasis:

1. **Reservoir Competence:** Some animals are better reservoir hosts for the Leishmaniinae parasites than others. Reservoir competence refers to the ability of an animal to harbour and facilitate vector infection. Animals that can maintain high levels of parasites in their blood and tissues for extended periods are considered more competent reservoir hosts.
2. **Parasite Load:** The number of parasites present in an infected animal's bloodstream is an important factor. Animals with higher parasite loads are more likely to transmit the parasite to sand flies during blood meals, contributing to the spread of leishmaniasis.
3. **Species Distribution:** The geographic distribution of an animal species is significant. If an animal species that carries *Leishmania* parasites is found in areas where the sand fly vector is present and human populations are at risk, the animal becomes a potential source of infection.
4. **Feeding Behaviour of Sand flies:** Some sand fly species preferentially feed on certain animal hosts. If a reservoir animal is the preferred host for a particular sandfly species, it can significantly contribute to the maintenance and spread of the disease.
5. **Ecological Niche:** Animals with overlapping ecological niches with humans are more likely to contribute to disease transmission. Urban and peri-urban animals that live close to human habitats can facilitate the transmission of parasites between animals and humans.
6. **Immune Response:** The immune response of an infected animal can influence the transmission of *Leishmania* parasites. Animals that mount weak immune responses may have higher parasite loads and are more likely to transmit the parasite to sand flies.
7. **Behavioural Factors:** Animals that exhibit behaviours such as communal nesting, close contact with humans or other animals, and frequent movement between habitats can facilitate the spread of parasites between hosts and increase the risk of transmission.
8. **Genetic Factors:** Genetic variations within animal populations can impact their susceptibility to *Leishmania* infection. Some animals may possess genetic traits that make them more or less likely to become infected and transmit the parasites.
9. **Host Longevity:** Animals with longer lifespans may contribute more to disease transmission, as they have more opportunities to encounter sandflies and be involved in the transmission cycle.
10. **Zoonotic Potential:** The zoonotic potential of a parasite refers to its ability to infect both animals and humans. Animals that carry *Leishmania* species that can infect humans are of higher concern due to the direct risk they pose to human health.

## INFECTIOUSNESS

Vector-borne diseases are transmitted to humans or animals by the bite of infected hematophagous arthropods, such as mosquitoes, ticks, fleas, sand flies and midges. A requirement of the host’s infection is that it must be infectious. Levels of infectiousness are influenced by various pathogen characteristics such as replication rate, incubation period, tissue tropism as well as the host’s sex. Assessing infectiousness is a crucial step in understanding and managing transmission dynamics. It requires a multidisciplinary approach that combines laboratory experiments, mathematical modelling, field studies, and genetic analysis.

Leishmaniasis is a vector-borne parasitic disease and follows the principal considerations that are the same in assessing the infectiousness of any vector-borne disease. The principal factors affecting infectiousness include its replication rate, incubation period and tissue tropisms. In the case of xenodiagnosis there may be variations in the number of flies infected that are related to the size of the blood meal and intrinsic susceptibility. This is particularly pertinent when the natural vector is not used.

The surge in the usage the polymerase chain reaction (PCR) in examining tissues from wild animals has led to a considerable increase in the records of Leishmaniinae in lizards, rodents, carnivores, bats, and primates. For example, two different PCR studies revealed *Leishmania* (*Leishmania*) *amazonensis* in 10 bat species from Brazil^14^ and *L.*(*L.*) *mexicana* in another 10 bat species from Mexico^15^. But what is the epidemiological importance of such infections and are they infectious to sand flies? This same question applies to all Leishmaniinae PCR records in wild animals and it could be interpreted that *Leishmania* DNA had been detected and not active infections.

## THE DEFINITIVE TEST OF INFECTIOUSNESS: XENODIAGNOSIS

An important diagnostic method for evaluating infectiousness introduced by Emile Brumpt^16^ is xenodiagnosis, which is feeding vectors on the infected animals. It has been used extensively since the early 1900s in the Old World^17^ and to a lesser degree in the Americas^18^, especially for parasites associated with cutaneous leishmaniasis. Sand fly colonization paved the way to determine infectiousness more objectively. The first phlebotomine xenodiagnosis with laboratory bred flies on a neotropical wild animal was in 1964^19^. It showed that an *Endotrypanum* infection of a two-toed sloth developed in laboratory reared *Lutzomyia sanguinaria.* This was followed in 1972^20^ when laboratory bred *Lu. gomezi* were fed on a wild two-toed sloth. *Leishmania* was isolated from1/10 fed flies. These two studies showed that sloths were infectious for both *Endotrypanum* and *L.* (*V.*) *panamensis*. 

Infectiousness of dogs and humans in different phases of their infections is fundamental in evaluating the feasibility of visceral leishmaniasis control. An in depth study using xenodiagnosis^21^ followed a cohort of naturally infected dogs in an endemic focus of visceral leishmanias in Marajó Island, Brazil. It showed that 7 highly infected dogs (17%) were responsible for over 80% of the infected sand flies. Although there was a positive correlation between the diagnostic tests and infectiousness, they did not detect infectious dogs in the lower latency period. The analysis of this data concluded that culling based on the diagnostic tests, that included PCR tests would not control the disease. In another study^22^ it was found that qPCR parasite loads in the skin and bone marrow of non-infectious dogs and foxes were similar indicating that in these two canids a positive qPCR does not reflect infectiousness. A problem with comparing tissue parasite loads with the qPCR is the standardisation. Calculating parasite loads based on tissue volume or weights is less reliable than using copies of a host gene, such glyceraldehyde-3-phosphate dehydrogenase (GAPDH)^23^. 

A critical question in the epidemiology of visceral leishmaniasis is are asymptomatic patients infectious? A study in Brazil^24^ found they were not. More recently a xenodiagnosis study in India^25^ also found that asymptomatic individuals were not infectious and that active visceral and post-kala-azar dermal leishmaniasis were. This led to the conclusion that the quest for markers of infectiousness may not be so important^26^. However, does this proposition apply to wild reservoir hosts? In our opinion it does not, as Leishmaniinae infections in their wild hosts are asymptomatic. Because of this finding a molecular test that evaluates infectiousness in wild animals is vital. Also, in multi-host reservoir situations the priority of one reservoir over another is relative to its infectiousness. Dogs should not be considered in the “wild animal category” as canine visceral leishmaniasis is a fatal disease.

## SEARCHING FOR A XENODIAGNOSIS SURROGATE USING EXPERIMENTAL INFECTIONS

Xenodiagnosis on man and dogs is difficult but logistically it is much more difficult to do this on wild hosts captured in endemic foci. An alternative is to investigate infectiousness of experimental infections in laboratory bred animals that have been found infected in the field. There are limitations to such experiments as normally two caveats associated with natural infections are lacking: infection by the vector and constant exposure to infected bites.

*L.* (*V.*) *braziliensis* infections have been found in rodent species such as *Rattus rattus, Nectomys squamipes, Necromys lasiurus, Holochilus scieurus* and *Sigmodon hispidus*^27^^,^^28^^,^^29^^,^^30^. The discoveries indicating that rodents were primary and secondary *L.* (*V.*) *braziliensis* reservoirs led to investigations of the infectiousness of experimental infections in laboratory bred *R. rattus, N. squamipes, Ne. lasiurus*^23^. The animals were infected by inoculating stationary phase promastigotes subcutaneously and intraperitoneally. Skin lesions occurred in 25.5% of the animals. They healed after 6 months, which was when xenodiagnosis with *Lu. longipalpis* were performed. All 18 *Ne*. *lasiurus* infected sand flies as did 10/18 *N. squamipes* and 6/18 *R. rattus.* The difference in the infectiousness of *Ne. lasiurus* to the other two rodents was statistically significant.

Parasite loads were evaluated in ear skin, spleen, and liver tissues by quantitative polymerase chain reaction (qPCR). The parasite loads were significantly lower in *R. rattus* but there was no similar difference between the other two species. Multivariate logistic analyses of the proportion of infected flies showed that *Ne. lasiurus* tended to be more infectious but there was no statistical difference between *N. squamipes* and R. *rattus.* There was a positive association between the skin log_10_ parasite loads and the differences between the number of infected flies between the species. However, what was surprising was that the even though the parasite loads were significantly lower in *R. rattus* its infectiousness was not significantly different from *N. squamipes*. 

The qPCR appeared to be a useful guide of assessing infectiousness bearing in mind that the infections were not initiated by sand fly bite. However, not all infections indicated by positive qPCRs were infectious to sand flies. It was noteworthy that significantly less ear infections occurred in *R. rattus* than Ne. *lasiurus* and *N. squamipes.* Importantly this study showed that after 6 months animals were still infectious. The finding of parasites in ear tissue and the fact that lesions appeared in the tails of some animals confirmed metastasis had occurred indicating that this is an innate characteristic of *L.* (*V.*) *braziliensis* irrespective of the host.

A similar course of infection to the one mentioned above has been noted for experimental infections^31^ of *L*. (*L*.) *major* in *Meriones shawi*. A mixture of sand fly saliva and parasites from the midgut were inoculated into the pinnae. What is very interesting in this study is that in certain percentage of jirds the parasites migrated to the other ear, the skin of the tail and paws and to the liver and spleen. In all 67% were infectious to *Phlebotomus papatasi,* some before the development of a lesion at the inoculation site. In some animals the pinnae lesions healed but remained swollen and in others they continued to be ulcerative. This contrasts with similar experiments in the great gerbil, *Psammomy*s *obesus*, in which ulceration and visceralization did not occur^32^. 

## SEARCHING FOR A XENODIAGNOSIS SURROGATE USING NATURAL INFECTIONS

A way of overcoming the limitations of studying infectiousness in laboratory bred reservoirs is to investigate it in natural infections and vectors. This was made possible during a longitudinal capture-mark-recapture (CMR) study of wild and synanthropic rodents naturally infected with *Leishmania (V.) braziliensi*s in northeast Brazil^10^. Over 27 months 603 rodents belonging to 8 species were re-captured on 1,051 occasions. The most abundant species were *N. squamipes* (41%)*, R. rattus* (25%) and *Ne. lasiurus.* (14%). 

 As animals were caught and released the most accessible tissue was blood and skin. Since there was no difference between the positivity of the internal organs and ear skin in the experimental studies^23^ the latter was chosen in the CMR study. A standard PCR using kDNA probes and qPCR were used to detect infections in these tissues. There was a highly significant difference between the two tests. The qPCR detected infections in 38.7% of the blood samples while the PCR only detected infections in 5.8% but only 1.8% of the ear samples were positive. The reasons for this are unknown but it be differences in metastases patterns of natural and experimental infections. 

Xenodiagnosis were performed in the field on 5 rodent species (*Akodon cursor, N. squamipes*, *Ne. lasiurus*, *Oxymycterus dasytrichus* and *R. rattus*) with *Nyssomyia whitmani* and *Lu. longipalpis*^10^*.* All the species were infectious to sand flies. Multiple *Ny. whitmani* xenodiagnosis trials on *N. squamipes* were positive in 34/41 trials on positive animals and in 7/41 trials on negative animals. The empirical probability of a *N. squamipes* with a positive molecular test being infectious was 0.83 while for one with a negative test it was 0.17. These results indicate that for molecular data to be relevant in relation to infectiousness it must be based on several observations before concluding the transmission threat of a population. There were a few animals whose molecular test was negative when first captured but whose xenodiagnosis was positive. When captured again both tests were positive. A possible explanation for this is that they represented skin infections that had not yet visceralized. This could explain the negative molecular tests in animals with positive xenodiagnosis. Overall, these results demonstrated a qualitative but not quantitative association between the number of positive flies and blood infections measured by qPCR.

Surprisingly the CMR study showed that an animal’s sex influences its infectiousness and should be considered when evaluating the validity of a potential xenodiagnostic molecular surrogate. Female *N. squamipes* were less infectious than males, but there was no significant difference between their infection rates.

The increasing awareness of host/vector specificity^33^^,^^34^^,^^35^ within the Leishmaniinae raises the question as to the validity of results when the natural vector is not used for xenodiagnosis. There are no flourishing colonies of ACL vectors, but a viable option is to use laboratory reared *Lu. longipalpis*. No significant differences were seen between xenodiagnosis with these flies and *Ny. whitmani* fed on *R. rattus.* Even though the mean qPCR value was greater for 3 *N. squamipes* significantly more *Lu. longipalpis* became infected when fed on 2 *R. rattus,* Further experiments are needed before saying that *Lu. longipalpis* detects *L. (V.) braziliensi*s infections more efficiently in *R. rattus* than *Ny. whitmani*, but there is a suggestion that this may be the case. However, it is safe to conclude that, *Lu. longipalpis* was as efficient as *Ny. whitmani* in detecting infections of *L. (V.) braziliensi*s and therefore can be used as surrogate for the natural vector. We emphasize that this only applies to *L. (V.) braziliensi*s in wild rodents and should not be interpreted as a generalization for detecting all Leishmaniinae species. Each species needs to be investigated individually with its natural host(s) and vector(s).

## DISCUSSION

The closer the empirical probability of infectiousness in comparing a potential molecular surrogate with xenodiagnosis results reaches the value of one the better. The greatest potential error with the molecular test, besides its sensitivity and specificity is examining tissues in which the parasites are not found. For instance, it is pointless to determine the parasite load in blood to evaluate the infectiousness of the hosts of *L.* (*L.*) *amazonensis*^36^ and *L.* (*V.*) *panamensis* as both are primarily skin parasites that rarely occur in the blood^20^. The difficulty in choosing the sampling site for evaluating infectiousness is the paucity of information on tissue tropisms of the different Leishmaniinae species in their natural hosts. This is also true for determining a parasite’s prevalence in a host population that relates directly to infectiousness.

A study of the infectiousness of mice experimentally infected with *L. (L.) donovani* showed that parasite distribution in the skin was patchy^37^. In the *L. (V.) braziliensi*s CMR study^10^ there was evidence of skin infections because of positive xenodiagnosis in animals whose blood qPCRs were negative. Also, in that study there was a poor relationship between ear skin positive qPCRs and positive xenodiagnosis suggesting the wrong site had been sampled. In mice infected with *L. (L.) donovan*i the inoculation site was found to remain positive for many months^38^. Following this finding it is possible that the most likely skin site containing parasites is where infected sand flies bite. Based on natural and experimental *L. (V.) braziliensi*s infections^10^^,^^23^ in rodents it appears that parasites migrate from the skin to the blood and viscera. Behavioural studies on sand fly feeding habits on wild animals would be helpful in locating where parasites are most likely to be found in the skin. 

Leishmaniinae infections in animals captured in the field are typically asymptomatic and there is overwhelming evidence that asymptomatic natural and experimental infections are infectious^10^^,^^23^^,^^31^^,^^39^. Skin lesions have been seen in some wild animals^36^^,^^40^. Local trauma has been found to provoke lesions in patients who are asymptomatic but have a history of cutaneous leishmaniasis^41^^,^^42^. When seen lesions in wild animals are on the tails and on the ears, both sites can easily be traumatized by accidents or fighting and are likely linked to forms of trauma that exacerbate an occult infection. However, the large number of parasites in such lesions could greatly increase infectiousness to sand flies. A percentage of gerbils infected with *L.* (*L*.) *major* have visible lesion on their ears and parasites can be detected in smears^43^. Microscopically 22% of the ear samples from *Rhombomys opimu*s, whereas 55% positive in a nested-PCR test showing a greater number of asymptomatic infections. 

The two most important sources of parasites for sand flies and midges are the blood and skin. If parasites are detected or isolated from blood the probability that the animal is infectious is high but not absolute. However, many records of Leishmaniinae infections in wild animals are based on molecular evidence of parasites in the spleen and liver. For instance *L*. (*L*.) *mexicana* was found in the skin of 15%, in the liver of 25% and in the spleen 45% of samples from wild rodents, but blood was not examined^44^. Without comparisons of viscera and blood parasite loads it is difficult to know how they might relate to infectiousness. This emphasises the need to have comparative data on parasite loads of the each Leishmaniinae species in different tissues. The qPCR appears to be the most sensitive method for doing this and is potentially a surrogate for xenodiagnosis. The level of concordance with sand fly xenodiagnosis results and a molecular test not only depends the tests sensitivity but also on the choice of tissue and the method used to standardize the parasite load. 

Accurate data on infectiousness in both symptomatic and asymptomatic animals are essential inputs for creating epidemiological models that can be used in assessing the potential impact of interventions and making informed decisions on resource allocation and response strategies A difficulty of designating an animal as being asymptomatic is in the choice of the clinical or molecular parameters^45^, particularly for such animals as dogs with visceral leishmaniasis. However, based on the lack of infectiousness of asymptomatic cases of kala-azar in India it has been suggested that “the elusive quest for markers of infectiousness might not be as important as previously thought”^26^. This is maybe a valid comment for asymptomatic visceral leishmaniasis in man and perhaps dogs, but not for Leishmaniinae infections in wild animals that are characteristically asymptomatic.
